# Customization of UWB 3D-RTLS Based on the New Uncertainty Model of the AoA Ranging Technique

**DOI:** 10.3390/s17020227

**Published:** 2017-01-25

**Authors:** Bartosz Jachimczyk, Damian Dziak, Wlodek J. Kulesza

**Affiliations:** 1 BetterSolutions S.A., Al. Grunwaldzka 472, 80-309 Gdansk, Poland; 2 Faculty of Electrical and Control Engineering, Gdansk University of Technology, G. Narutowicza 11/12, 80-233 Gdansk, Poland; damian.dziak@pg.gda.pl; 3 Department of Applied Signal Processing, Blekinge Institute of Technology, 37179 Karlskrona, Sweden; wlodek.kulesza@bth.se

**Keywords:** accuracy and precision, angle of arrival, correction vector, indoor localization systems, real-time locating systems

## Abstract

The increased potential and effectiveness of Real-time Locating Systems (RTLSs) substantially influence their application spectrum. They are widely used, inter alia, in the industrial sector, healthcare, home care, and in logistic and security applications. The research aims to develop an analytical method to customize UWB-based RTLS, in order to improve their localization performance in terms of accuracy and precision. The analytical uncertainty model of Angle of Arrival (AoA) localization in a 3D indoor space, which is the foundation of the customization concept, is established in a working environment. Additionally, a suitable angular-based 3D localization algorithm is introduced. The paper investigates the following issues: the influence of the proposed correction vector on the localization accuracy; the impact of the system’s configuration and LS’s relative deployment on the localization precision distribution map. The advantages of the method are verified by comparing them with a reference commercial RTLS localization engine. The results of simulations and physical experiments prove the value of the proposed customization method. The research confirms that the analytical uncertainty model is the valid representation of RTLS’ localization uncertainty in terms of accuracy and precision and can be useful for its performance improvement. The research shows, that the Angle of Arrival localization in a 3D indoor space applying the simple angular-based localization algorithm and correction vector improves of localization accuracy and precision in a way that the system challenges the reference hardware advanced localization engine. Moreover, the research guides the deployment of location sensors to enhance the localization precision.

## 1. Introduction

The continuing development of wireless technologies has led to an enhancement of the capabilities and efficiency of Real-time Locating Systems (RTLSs), which are a particular example of Indoor Locating Systems (ILSs). RTLSs have the ability to define the position of an item anywhere in a defined space at a point in time that is, or is close to a real time [1]. Ultra-wideband (UWB) is a short-range and energy efficient radio technology useful in a high-bandwidth wireless communication. Due to their functionalities and performances, the use of UWB-based RTLSs as tracking management systems has gained increasing attention in industrial and logistic applications, for instance container terminals [2], and warehouses [3]. Besides that, RTLSs have been widely applied in security applications in construction sites as a safety system [4,5], in healthcare for precisely monitoring and tracking people and goods [6,7,8] and in agriculture for animal behaviour monitoring [9].

The UWB-based indoor RTLSs are able to estimate a target’s location with high accuracy and precision, which depend, inter alia, on the working environment, system architecture, and localization algorithm. The estimation process uses ranging techniques, which vary for different signals such as Received Signal Strength (RSS), Time of Arrival (ToA), Time Difference of Arrival (TDoA) and Angle of Arrival (AoA) [10]. Some commercial UWB-based RTLSs use hybrid localization methods, however the performance improvement is mitigated by the system price and complexity.

The AoA-based ranging technique is commonly used in UWB-based RTLSs. This technique uses direction-sensitive antennas as location sensors to estimate the direction of the RF signal from a tag [11]. The disadvantage of the AoA approach, compared with e.g., the TDoA solution, is its accuracy. It is counterpoised by the system’s simplicity, which leads to a lower price and simplicity of real time implementation. The customizations of the AoA-based RTLS for different working environments help to overcome the accuracy disadvantage by improving the efficiency and localization performance. The performance assessment and analytical uncertainty model of AoA localization for the given environmental characteristics and RTLS’s architecture are needed for the customizations.

This paper focuses on an improvement of the system’s performance by applying the analytical customization method for different working environments. The proposed approach is based on the RTLS’s performance assessment using the new analytical uncertainty model of AoA localization in a 3D indoor space. The uncertainty model comprises two localization performance measures: accuracy and precision. Furthermore, the paper introduces the angular-based 3D localization algorithm combining the ranging technique and extrapolation method.

An implementation of the proposed customization method of UWB-based RTLS has been verified and shows an accuracy improvement of 55%. The used analytical model of uncertainty in terms of precision has been validated and proved the entire matching with the experimental results. The analytical and experimental evaluation of the performance of the proposed AoA-based localization algorithm has been applied for different LSs configurations in the given test environment. The comparison of the AoA-based localization results with the commercial RTLS’s algorithm results confirms the expediency of the proposed approaches.

## 2. Survey of Related Works

Due to their localization capabilities and reliability, UWB-based RTLSs, as a kind of wireless RF-based ILSs, are widely used in the industrial sector, but also in other logistic and security applications. These systems are able to localize a target with accuracy up to several centimetres in an indoor space. The performance of these systems highly depends on their architecture, LSs arrangement, and the location engine [12].

Depending on the application character and system structure, localization platforms apply a variety of ranging techniques such as RSS, ToA, TDoA, AoA [13], along with different position estimation algorithms such as trilateration, triangulation, fingerprinting, dead path reckoning, and some others [11]. The most basic ranging technique is based on the power measure of RF, called RSS. However, the RSS methods are highly affected by obstacles and multipath fading, along with environmental interferences [14,15]. Therefore, currently RTLSs use the RSS rather as a complementary measure, for instance in data filtration [16], and the other measures like ToA/TDoA and AoA are more commonly used in position determination [13].

Due to their popularity, ToA and TDoA-based estimation algorithms are widely researched. In [17], the authors discuss the main sources of errors in the ToA-based ranging in the multipath environments. These include the multipath fading and direct path excess delay, but also blockage, narrowband and multi-user interferences and clocks drift. The authors of [18] present a quality-enhancing and novel ToA-based ranging scheme with an improved detection of distorted UWB pulses in dense environments, such as a residential office or factory. Selimis et al. propose and evaluate in a real scenario the ToA-based estimation algorithm consisting of an improved acquisition unit, which detects and synchronizes UWB pulses. The acquisition unit applies a single peak identification procedure, which detects the strongest multipath component of the signal using the channel impulse ratio estimation [19]. Shen et al. focus on ToA technology for range-based localization in UWB sensor networks [20]. The authors apply the Constant False Alarm Rate adaptive algorithm, which is based on the detection theory applied to radar systems. They also propose the Maximum Probability Detection method estimating the ToA by finding the maximum probability of the multipath signal components.

Range-based AoA approaches, also commonly used in RFID and UWB-based RTLSs, apply direction-sensitive antennas as location sensors to estimate the direction (the angle of arrival) of the signal from a tag [11]. The performance of AoA-based estimation algorithms are studied among others in [21,22,23]. Kim et al. propose an improvement of the AoA-based RTLS designed for Non-line-of-sight (NLoS) environments. The proposed algorithm, enhancing the estimation of the target’s position, applies a Dual Indirect Kalman Filter and weight filters [24]. In [25], the authors suggest a localization algorithm, which utilizes a biased estimation technique to increase the system performance. Moreover, the authors apply a statistical calibration method to improve the localization quality. Zampella et al. propose the sensor fusion of UWB RTLS with Inertial Measurement Unit (IMU) mounted in a mobile phone [26]. They combine the commercial UWB RTLS algorithm with the dead path reckoning estimation algorithm to carry out the localization both indoor and outdoor around a building. The performance analysis of AoA-based localization systems is studied in [27,28]. Using kinds of Fisher Information Matrices, the authors determine the optimal configuration of sensors to enhance the angle-related information in 3D space. An optimal configuration of sensors depends inter alia on the intensity of the measured noise, configuration constraints and the probabilistic distribution that defines the prior uncertainty in the target position.

The performance assessment of RTLSs can apply different physical measures depending on the type of RTLS and application. In [4], the authors investigate the performance in terms of accuracy, precision and reliability of an UWB-based RTLS tracking of multiple tags simultaneously. Furthermore, they analyse the impact of a number of receivers on localization performance under common conditions of construction sites i.e., occurrences of metal surfaces. Silva et al. evaluate the UWB-based Symmetrical Double Sided Two-way Ranging RTLS, which does not require synchronization [29]. They assess the indoor localization performance in terms of precision, accuracy, refresh rate and reliability in Line of Sight (LoS) and NLoS scenarios.

The measurement uncertainty of AoA may be evaluated using the measure of accuracy and precision, and the quality of the localization estimates of AoA measurements is an issue of several papers. It is proved that a type and characteristic of antenna [30], their deployment [31] and also a type of indoor environment significantly affect the measurement precision [12]. To reduce impacts of these factors, Crespo et al. perform the experimental study of different types of RTLS antennas in different indoor environments and find out the matching conditions [32].

The precision of AoA measurement uncertainty is related with the concept of Dilution of Precision (DoP), originally used in satellite navigation systems. Among others, Dempster et al. apply the DoP measure to characterize the quality of AoA-based positioning systems [33]. Arafa et al. investigate an effect of DoP on the localization performance of the optical wireless ILS [34]. In [12], the authors propose an analytical DoP-based uncertainty model of precision and apply it with the fingerprinting method to customize the RTLS aimed in an improvement of its performance in terms of localization precision.

The localization uncertainty in terms of accuracy can be characterized as a systematic error whose influence may be mitigated by an appropriate identification and compensation. Junhuai et al. in [35] propose a localization algorithm based on region divisions and error compensation to enhance the localization accuracy. Their algorithm divides the localization area into many sub-regions and a specific propagation model is defined for each sub-region. In [36], the authors show the influence of the calibration tag’s placement on AoA measurement uncertainty. They propose a suboptimal criterion how to allocate the calibration emitter in relation to sensors’ positions in an indoor space. Ghazaany et al. in [37] investigate how a mutual coupling compensation matrix influences the performance of the AoA-based small size uniform circular array. The authors propose a complex compensation matrix corresponding to the coupling effect between antennas’ elements. Myong et al. show an impact of signal interferences on the location accuracy of RFID-based RTLS in multipath environments [38]. The authors consider direct and indirect path components with time and phase delay differences.

## 3. Problem Statement and Main Contribution

From the review of related works, one can notice that localization methods for RTLSs perform differently. The accuracy, precision, processing time, cost and simplicity are the main measures of the system performance. Then, the challenge is to find out trade-offs of various aspects of the system’s performance for different methods. Furthermore, there are many factors influencing the performance of an indoor 3D localization, inter alia the system architecture, working environment, possible interferences etc. Most of the localization algorithms estimating the tag’s position in the indoor environment use ranging techniques, which are based on different measurement signals such as RSS, TDoA, AoA. Among these solutions, the angle-based (AoA ranging technique) localization algorithms show a big potential of performance improvement, especially in 3D applications. Therefore, one of the possible enhancement approaches is to develop a measurement analytical model, which can facilitate the customization of the AoA ranging technique in different 3D environments.

The main objective of this paper is to develop and then to implement in a simulation environment the geometrical (analytical) uncertainty model of AoA localization in a 3D indoor space, while considering the localization uncertainty in terms of accuracy and precision, which extends and enhances the uncertainty model proposed in [12]. The goal of modelling is to determine the efficient system configuration. Furthermore, the proposed model facilitates the system customization by defining and implementing the correction vectors for different working environments in order to improve the system’s performance in terms of its accuracy. Additionally, the angular-based 3D localization algorithm, which estimates the tag’s position in 3D from interfered measurement signals of azimuth and elevation angles, is introduced.

The main contribution of this paper is an improvement method of system’s performance by applying the analytical customization for different working environments. The method is based on a new holistic approach to localization uncertainty in terms of precision and accuracy, and defining a geometrical model of the AoA localization method in 3D. The model facilitates an uncertainty analysis of the AoA ranging technique. The analytical model is implemented in Matlab and used to show how different the system’s features influence its performance. The measurement precision model is verified by analysing the matching ratio of the simulated and experimental results. To show the accuracy enhancement, by applying the correction vector, the proposed customization of the system for a given working environment is validated by physical experiment. The reference RTLS localization algorithm is applied to verify that the customization of AoA ranging techniques can challenge the advances in hardware of the UWB-based RTLS technology.

## 4. RTLS’s Performance Assessment

The assessment of RTLS’s performance is necessary to find trade-offs among different technologies and methods. In general, the assessment needs to be considered separately for static and dynamic localization modes [4,39]. In the static mode, it can be evaluated using different measures inter alia localization uncertainty, sensitivity [9] and response time. In the dynamic mode, these measures are affected by the speed of a tag, a number of tags, and the complexity of their paths.

**Figure 1:**
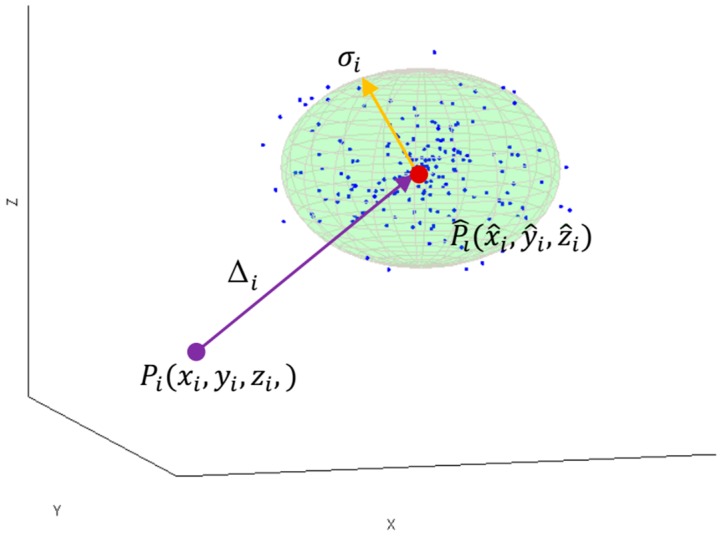
Illustration of 3D localization accuracy and precision for *i*-th tag’s position.

The simplified interpretation of 3D localization uncertainty in terms of accuracy and precision measures at the true tag’s position $P_{i}$ in coordinates $\left(x_{i},y_{i},z_{i},\right)$ is illustrated in Figure 1.

The accuracy measure $\Delta_{i}$ represents the distance between the true position $P_{i}$ and the location estimate $\hat{P_{i}}$ obtained from the RTLS, whereas, the precision is illustrated by the sphere with an estimate’s standard error $\sigma_{i}$ as a radius. The sphere is centered in the estimated position $\hat{P_{i}}$ and includes respectively 68% of *N* measured localization samples. Both localization uncertainty measures: accuracy and precision characterized dispersion of measured results from the tag’s true position. In the following sections, the localization performance is modelled in a static mode using the two measures: accuracy and precision [40].

### 4.1. Accuracy

The accuracy property expresses the capability to obtain the true value of a measurand [40]. The mean uncertainty component, the localization accuracy, $\Delta x_{i}$ of the *x* coordinate estimated for the point $P_{i}$ located in the test environment, can be expressed as:

(1) $$\Delta x_{i}={\hat{x}}_{i}-x_{i}$$

where variables of $P_{i}\left(x_{i},y_{i},z_{i}\right)$ respectively refer to the true localization coordinates at *i*-th position of the tag, whereas variables of $\hat{P_{i}}\left({\hat{x}}_{i},{\hat{y}}_{i},{\hat{z}}_{i}\right)$ refer to the mean of *N*-th times measured localization coordinates at *i*-th tag’s position. If the remaining two mean uncertainty components $\Delta y_{i},\;\mathrm{and}\;\Delta z_{i},$ of *y* and *z* coordinates respectively are specified analogically to Equation (1), then the localization uncertainty $\Delta_{i}$ at *i*-th tag’s position can be shown as follows:

(2) $$\Delta_{i}=\sqrt{\Delta {x_{i}}^{2}+\Delta {y_{i}}^{2}+\Delta {z_{i}}^{2}}$$

### 4.2. Precision

The second uncertainty measure, the localization precision, describes the measurement’s repeatability and is based on an estimate of the mean standard error $\bar{\sigma }$ of the mean localization uncertainty. A low value of the standard error means high precision and vice versa. For the *i*-th tag’s position, which is estimated from *N* measurements, a standard error of the mean (SEM) $\bar{\sigma_{x}}$ of the component *x*, can be expressed in relation to its variance as:

(3) $${\bar{\sigma }}_{x_{i}}=\sqrt{\frac{\sigma_{x_{i}}^{2}}{N}},$$

where ${\bar{\sigma }}_{x_{i}}$ and $\sigma_{x_{i}}^{2}$ are SEM and variance respectively of the *x* component at *i*-th tag’s position. The remaining two SEMs ${\bar{\sigma }}_{y_{i}}\;\mathrm{and}\;{\bar{\sigma }}_{z_{i}}$ of *y* and *z* coordinates respectively can be described analogically. Then the corresponding SEM ${\bar{\sigma }}_{i}$ of the localization estimate of *i*-th tag’s position can be calculated as follows:

(4) $${\bar{\sigma }}_{i}=\sqrt{{{\bar{\sigma }}_{x_{i}}}^{2}+{{\bar{\sigma }}_{y_{i}}}^{2}+{{\bar{\sigma }}_{z_{i}}}^{2}}$$

## 5. The AoA-Based Localization Algorithm

The angular-based localization algorithms of RTLSs use angles of arrival of the radio wave, measured by a pair of LSs. For each measurement cycle, to determine the tag’s location, AoA ranging and extrapolation procedures are processed one by one. In [12], the simplified 2D AoA localization algorithm using azimuth angles is presented. In the following subsection, a specific model of ranging technique using azimuth and elevation angles for the 3D localization is proposed. Moreover, the tag’s location is estimated based on an extrapolation technique described in the second subsection of this chapter.

### 5.1. AoA Ranging Technique

The ranging technique, described in this section, is illustrated by the *AB* pair of LSs. The initial installation stage of the ranging technique procedure is performed only once, at the beginning of measurement process when the workspace and LSs’ coordinates are defined and the system is calibrated. Then, from the installation data the workspace geometry and the coordinates, $A\left(x_{A},y_{A},z_{A}\right)$ and $B\left(x_{B},y_{B},z_{B}\right)$ of the active LSs’ positions, are established. Moreover, the calibration procedures for azimuth and elevation angles are performed. The calibration procedure of UWB-based RTLS for azimuth angles is described in [12] and the calibration procedure for elevation angles looks similarly. Therefore, after the initial phase, the workspace geometry, LSs’ coordinates, and even the calibration angles and lines are defined.

**Figure 2:**
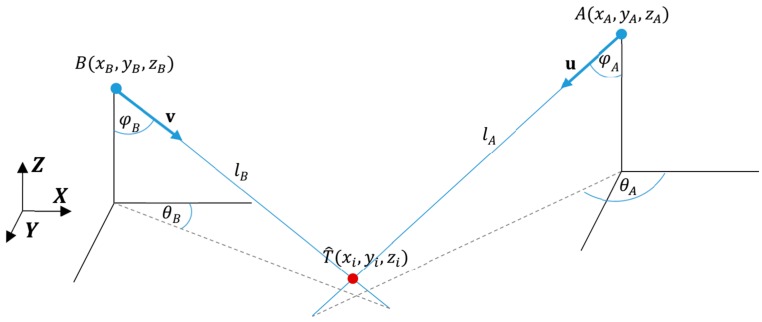
Graphical interpretation of the 3D AoA ranging technique.

The basic stage of the AoA ranging technique is performed in each measurement cycle when the directions of the arrival paths from the tag to the two LSs are measured. Each LS consisting of antenna array element measures the direction of the receiving tag’s radio wave as azimuth and elevation angles of arrival. Radio waves take the form of UWB pulses with very short durations, which are emitted by the tag. A geometrical interpretation of 3D AoA ranging techniques for a pair of LSs *AB* is illustrated in Figure 2. The line $l_{A}$ represents the arrival path, which passes through the tag’s position $\hat{T}$ and LS_A_’s coordinates $A\left(x_{A},y_{A},z_{A}\right)$. The line is projected perpendicularly onto the *XY* plane, called reference plane. The azimuth angle $\theta_{A}$ is the angle between the line projected on the reference plane and the axis *X*. The elevation angle $\varphi_{A}$ is the angle between the line $l_{A}$ and the axis *Z*. Azimuth and elevation angles measured by the LS_B_ are defined analogically.

The lines $l_{A}$ and $l_{B}$ can be represented by direction vectors u and v with initial points in A and B respectively. Both u and v direction vectors are computed from measured azimuth angles $\theta_{A}$ and $\theta_{B}$ and elevation angles $\varphi_{A}$ and $\varphi_{B}$ respectively. Then the vectors between the LSs placed in *A* and *B* and the tag are depicted as:

(5) $$\lambda_{A}u=\lambda_{A}\left[\begin{array}{c} u_{x}\\ u_{y}\\ u_{z} \end{array}\right]=\lambda_{A}\left[\begin{array}{c} \sin\varphi_{A}\cdot \cos\theta_{A}\\ \sin\varphi_{A}\cdot \sin\theta_{A}\\ \cos\theta_{A} \end{array}\right]$$

(6) $$\lambda_{B}v=\lambda_{B}\left[\begin{array}{c} v_{x}\\ v_{y}\\ v_{z} \end{array}\right]=\lambda_{B}\left[\begin{array}{c} \sin\varphi_{B}\cdot \cos\theta_{B}\\ \sin\varphi_{B}\cdot \sin\theta_{B}\\ \cos\theta_{B} \end{array}\right]$$

where $\lambda_{A}$ and $\lambda_{B}$ are length parameters of the distance vectors, and $u_{x}$, $u_{y},\;u_{z}$ and $v_{x}$, $v_{y},\;v_{z}$ are direction vectors’ u and v components respectively. Both direction vectors u and v along with the length parameters $\lambda_{A}$ and $\lambda_{B}$ and the coordinates of the initial points *A* and *B* determine the end-points $T_{A}$ and $T_{B}$ of distance vectors $\lambda_{A}u$ and $\lambda_{B}v$ respectively. Therefore, resultant points $T_{A}$ and $T_{B}$, which estimate the tag’s location are depicted as:

(7) $$T_{A}=A+\lambda_{A}u^{-1}$$

(8) $$T_{B}=B+\lambda_{B}v^{-1}$$

In an ideal case, the tag’s position $\hat{T}$ is defined by $T_{A}=T_{B}$, since both points should have the same coordinates. Using Equations (7) and (8), a set of three separate linear functions is formed and the coordinates of the tag’s position $\hat{T}\left(x_{T},\;y_{T},\;z_{T}\right)$ along with the two parameters $\lambda_{A}$ and $\lambda_{B}$ can be calculated.

### 5.2. AoA Extrapolation Method

**Figure 3:**
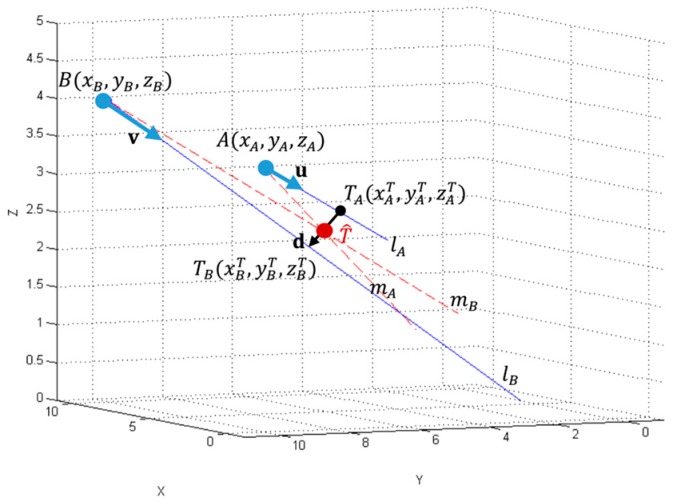
Extrapolation of localization point from the distance of the askew lines.

In general, due to the uncertainty of AoA measures, the arrival paths represented by the lines $l_{A}$ and $l_{B}$ for LS_A_ and LS_B_ respectively are askew and there is no intersection point, as shown in Figure 3.

Then, the lines $l_{A}$ and $l_{B}$ need to be extrapolated to lines $m_{A}$ and $m_{B}$ respectively, which intersect in the extrapolated tag’s position $\hat{T}$ [41]. The extrapolated solution can be defined as the middle of the shortest distance between the lines $l_{A}$ and $l_{B},$ which can be calculated using formula:

(9) $$d\left(l_{A},\;l_{B}\right)=\underset{T_{A}\epsilon l_{A},\;T_{B}\epsilon l_{B}}{\min}d\left(T_{A}^{T},T_{B}^{T}\right).$$

The shortest distance between the $\mathrm{lines}\;l_{A\;}\mathrm{and}\;l_{B}$ can be represented as a vector d with the initial point’s coordinates $T_{A}^{T}\left(x_{A}^{T},\;y_{A}^{T},\;z_{A}^{T}\right)$ and the end point’s coordinates $T_{B}^{T}\left(x_{B}^{T},y_{B}^{T},z_{B}^{T}\right)$. Vector d is perpendicular to both direction vectors u and v defined in the previous section. The extrapolated tag’s position $\hat{T}$ with coordinates $\left(x_{T},\;y_{T},\;z_{T}\right)$ is calculated as a midpoint of the vector d using the following formula:

(10) $$\hat{T}\left(x_{T},\;y_{T},\;z_{T}\right)=\left(\frac{x_{A}^{T}+x_{B}^{T}}{2},\frac{y_{A}^{T}+y_{B}^{T}}{2},\frac{z_{A}^{T}+z_{B}^{T}}{2}\right)$$

The extrapolated tag’s position $\hat{T}$ and two extrapolated arrival paths represented by lines $m_{A}$ and $m_{B}$ intersecting at the tag’s position $\hat{T}$ are used for the localization uncertainty modelling presented in the next section.

## 6. Modelling of Uncertainty of AoA-Based Localization

An uncertainty model of AoA localization in 3D is needed to customize the RTLS in different working environments to improve its performance. In [12] the localization uncertainty was represented by precision, sufficient for the applied enhancement method. However, the AoA localization uncertainty model might be upgrades with accuracy. For a specified RTLS architecture and working indoor environment, the model of localization measurement accuracy, defined here as an offset error, is presented in the first part of this chapter. The localization precision defined using a geometrical approach is introduced in the second subsection of this chapter.

### 6.1. Modelling of Offset Error and Correction Vector

For a specified UWB-based RTLS architecture used in a given working indoor environment, the uncertainty model of an offset error can be established heuristically. The offset error for a single measured position is systematic. However, for the given working environment consisting of a set of measured positions, the offset error consists of two components: random and systematic. Among others, the systematic component, which is constant for the whole environment, can be caused by the uncertainty of an initial calibration procedure when the calibration axes are defined in relation to the LSs’ positions. Whereas, the random component, varying at different localizations can be an effect of environmental characteristics specific at a given position. Another cause of the offset error can be heterogeneity of tags’ characteristics.

An effect of the localization offset error can be reduced by a correction vector, which depends on the system’s architecture and test environment, and is to be estimated heuristically. It may include the following sources:

- AoA LSs deployment measurement [30],
- AoA sensors array [42],
- tags’ characteristics,
- calibration process.

In a given environment, the correction vector for a certain pair of LSs, may be estimated based on measurements from *k* tags each sampled *N* times at *M* locations. For these numbers of tags, locations and samples, the *x* component of the correction vector, $kv_{x},$ may be calculated from:

(11) $$kv_{x}=\frac{1}{K\cdot M\cdot N}\overset{M}{\underset{m=1}{\sum }}\overset{N}{\underset{n=1}{\sum }}\overset{K}{\underset{k=1}{\sum }}\Delta x_{k,n,m}$$

where $\Delta x_{k,m,n}$ is the difference between the true localization and the *n*-th measurement at *m*-th localization of *k*-th tag. The remaining two correction vector components $kv_{y}\;\mathrm{and}\;kv_{z},$ of *y* and *z* coordinates respectively can be calculated analogically to Equation (11). Then the correction vector kv specified for the localization system in the test environment can be expressed as:

(12) $$kv=\left[\begin{array}{c} kv_{x}\\ kv_{y}\\ kv_{z} \end{array}\right]$$

### 6.2. Modelling of the Precision of 3D Localisation Based on AoA Technique

The precision model applied to the AoA ranging technique in 2D is described in [12]. The presented geometrical model is based on the azimuth component of AoAs. Since it was shown, that the pairs located on the shorter sides of the workspace ensure the most precise AoA-based localization [12], therefore, the following description concerns the pair LS_A_ and LS_B_ placed in the fixed positions $A\left(x_{A},y_{A},z_{A}\right)$ and $B\left(x_{B},y_{B},z_{B}\right)$ on the shorter side of the workspace. The specified tag T is located at the position $T_{i}\left(x_{i},y_{i},z_{i}\right).$ However, the selected configuration does not limit the model’s versatility.

For the LSs pair *AB*, each tag’s position on the workspace is defined by a set of two azimuth angles and a set of two elevation angles. The first set consists of vectors $AoA_{A}^{\theta }$ and $AoA_{B}^{\theta }$ of azimuth angles of arrival $\theta_{Ai}$ and $\theta_{Bi}$ measured by LSs *A* and *B* respectively. Analogically, elevation angles of arrival $\varphi_{Ai}$ and $\varphi_{Bi}$, measured by LSs *A* and *B* respectively, are represented by vectors $AoA_{A}^{\varphi }$ and $AoA_{B}^{\varphi }$, which constitute the second set.

Each measurement is repeated *N* times and using the ranging and extrapolation techniques, then N samples of *i*-th tag position $T_{i}$ are estimated as intersections (10) of lines $m_{A}$ and $m_{B}$ corresponding to the extrapolated paths of arrival. Based on experimental data, we assume that the distribution functions of azimuth and elevation measurements are normal. Then, at confidence level of 68.3%, the azimuth angles $\theta_{Ai}$ and $\theta_{Bi}$ are within the ranges $\theta_{Ai}\in \left(\bar{\theta_{A}}-{\bar{\sigma }}_{A}^{\theta },\;\bar{\theta_{A}}+{\bar{\sigma }}_{A}^{\theta }\right)$ and $\theta_{Bi}\;\in \left(\bar{\theta_{B}}-{\bar{\sigma }}_{B}^{\theta },\;\bar{\theta_{B}}+{\bar{\sigma }}_{B}^{\theta }\right)$ respectively, where the mean values $\bar{\theta_{A}}$ and $\bar{\theta_{B}}$ represent the best estimate of azimuth angles and $\pm {\bar{\sigma }}_{A}^{\theta }$ and $\pm {\bar{\sigma }}_{B}^{\theta }$ depict the SEMs of the azimuth AoA for LS_A_ and LS_B_ respectively. These ranges define the precision of the azimuth angle measurement called azimuth Angle of Precision, (AoP). Per analogy, the elevation angles $\varphi_{Ai}$ and $\varphi_{Bi}$ are within the ranges $\varphi_{Ai}\;\in \left(\bar{\varphi_{A}}-{\bar{\sigma }}_{A}^{\varphi },\;\bar{\varphi_{A}}+{\bar{\sigma }}_{A}^{\varphi }\right)$ and $\varphi_{Bi}\;\in \left(\bar{\varphi_{B}}-{\bar{\sigma }}_{B}^{\varphi },\;\bar{\varphi_{B}}+{\bar{\sigma }}_{B}^{\varphi }\right)$ respectively, which define the precision of the elevation angles measurement. The mean values $\bar{\varphi_{A}}$ and $\bar{\varphi_{B}}$ represent the best estimate of elevation angles, which are used to calculate the best estimate of the tag’s position $\hat{T}$. SEMs of the elevation AoA for both LS_A_ and LS_B_ are represented by $\pm {\bar{\sigma }}_{A}^{\varphi }$ and $\pm {\bar{\sigma }}_{B}^{\varphi },$ respectively. Thus, the normal distributions of the azimuth and elevation AoA of N samples for location sensor LS_A_ can be defined as $N_{A}^{\theta }\left(\bar{\theta_{A}},{\bar{\sigma }}_{A}^{\theta }\right)$ and $N_{A}^{\varphi }\left(\bar{\varphi_{A}},{\bar{\sigma }}_{A}^{\varphi }\right)$ respectively [27]. The probability distributions of the azimuth and elevation AoA of the set of N samples for location sensor *LS_B_* can be described analogically as $N_{B}^{\theta }\left(\bar{\theta_{B}},{\bar{\sigma }}_{B}^{\theta }\right)$ and $N_{B}^{\varphi }\left(\bar{\varphi_{B}},{\bar{\sigma }}_{B}^{\varphi }\right)$ respectively.

**Figure 4:**
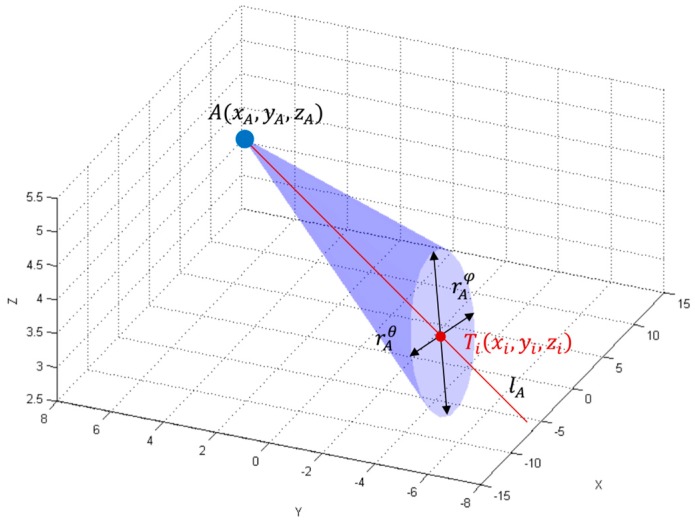
Illustration of uncertainty elliptical cone.

The LS_A_ is characterized by the elevation and azimuth AoPs, which can be used to form an elliptic cone representing the measurement precision in 3D, as shown in Figure 4. The cone’s vertex is placed at the LS’s position, $A\left(x_{A},y_{A},z_{A}\right)$ and its axis refers to the mean path of arrival $\bar{l_{A}},$ and its base at the estimated tag’s position is an ellipse with the axes defined by $r_{A}^{\theta }$ and $r_{A}^{\varphi }$ as follows:

(13) $$r_{A}^{\theta }=2\cdot \sqrt{{\left(x_{i}-x_{A}\right)}^{2}+{\left(y_{i}-y_{A}\right)}^{2}+{\left(z_{i}-z_{A}\right)}^{2}}\cdot \tan^{-1}{\bar{\sigma }}_{A}^{\theta },$$

(14) $$r_{A}^{\varphi }=2\cdot \sqrt{{\left(x_{i}-x_{A}\right)}^{2}+{\left(y_{i}-y_{A}\right)}^{2}+{\left(z_{i}-z_{A}\right)}^{2}}\cdot \tan^{-1}{\bar{\sigma }}_{A}^{\varphi }.$$

From the equations, one can see that the size of the elliptic base depends on SEM of azimuth ${\bar{\sigma }}_{A}^{\theta }$ and elevation ${\bar{\sigma }}_{A}^{\varphi }$ angles measurements and the distance from the active LS’s position to tag’s position $T_{i}$. Therefore, since the distribution functions of the measurement precision of azimuth and elevation AoAs are normal distribution functions, representing the dispersion of the path of arrival of N samples, then the surface area of the elliptic cone’s base at a given distance from the LS represents the bivariate SEM. The true localization occurs there with the confidence level of 68.2%.

Per analogy, the uncertainty of LS_B_ is characterized by an elliptic cone with the vertex in LS’s position $B\left(x_{B},y_{B},z_{B}\right)$ and the axis refers to the mean path of arrival ${\bar{l}}_{B}$. The cone’s base at the estimated tag’s position is an ellipse, with the axes defined by $r_{B}^{\theta }$ and $r_{B}^{\varphi },$ respectively.

**Figure 5:**
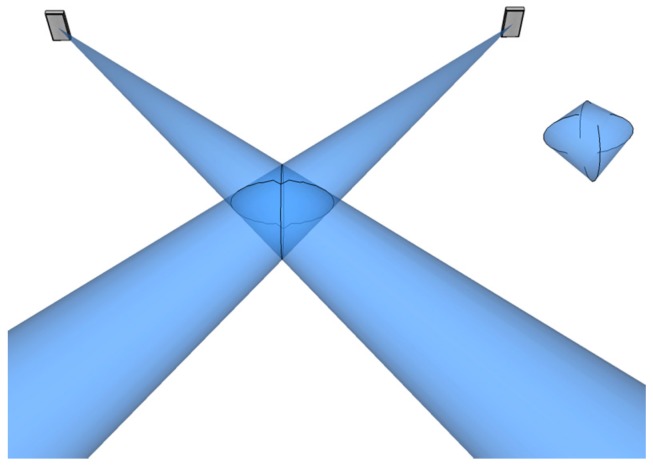
Illustration of uncertainty cones and the solid—common part of the two cones.

The two elliptical cones with vertices at the active LSs’ positions $A\left(x_{A},y_{A},z_{A}\right)$ and $B\left(x_{B},y_{B},z_{B}\right)$ represent the precision of tag’s AoA measurements. The cones cross each other and their common part forms the solid where the tag is truly located with the 46.5% probability corresponding to the product of two SDs probabilities, see Figure 5. The volume of the solid depends on the:

- precision of azimuth AoA measurements represented by SEM of ${\bar{\sigma }}_{A}^{\theta }$ and ${\bar{\sigma }}_{B}^{\theta }$ for LS_A_ and LS_B_ respectively,
- precision of elevation AoA measurements represented by SEM ${\bar{\sigma }}_{A}^{\varphi }$ and ${\bar{\sigma }}_{B}^{\varphi }$ for LS_A_ and LS_B_ respectively,
- distances from LSs positions $A\left(x_{A},y_{A},z_{A}\right)$ and $B\left(x_{B},y_{B},z_{B}\right)$ to the estimated tag’s position $\hat{T}$.

The volume of the solid is a measure of the uncertainty-precision of 3D AoA ranging technique. Its shape depends on the tag’s location in the test environment. The result of the cones’ penetration is presented in Figure 5. This is a particular symmetrical case when both cones have the same shape since azimuth and elevation AoP values are equal, i.e., elevation angles $\varphi_{A}$, $\varphi_{B}$ and azimuth angles $\theta_{A}$ and $\theta_{B}$ are equal.

**Figure 6:**
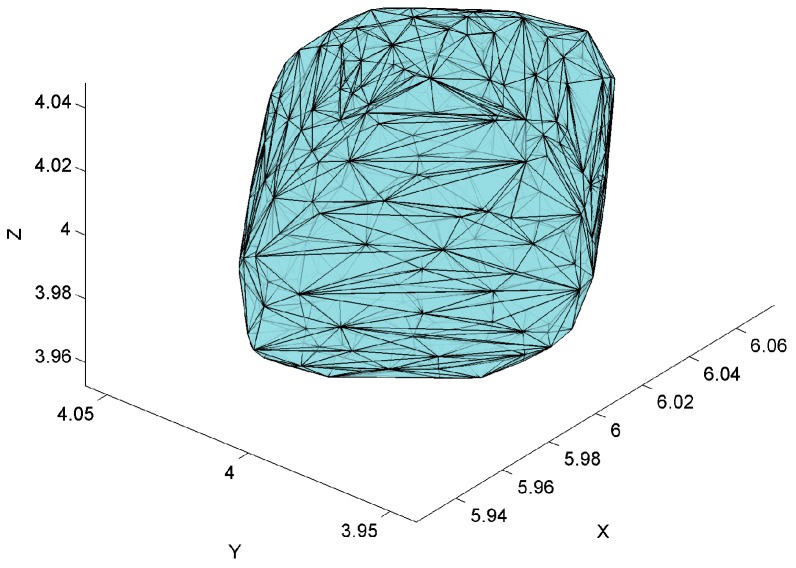
Convex hull of the points representing the solid.

The volume of the solid is calculated numerically using the 3D Delaunay triangulation computing method called Delaunay tetrahedralization [43]. The algorithm calculates the volume of the solid based on the sum of each individual tetrahedral volume. The boundaries of the 3D Delaunay triangulation represent the convex hull of the points set as shown on Figure 6. The shape of the convex hull matches the theoretical solid shape presented in Figure 5.

## 7. Precision Model Evaluation

The implementation of the proposed 3D model is done in Matlab 2014b with the Signal Processing, Optimization and Computational Geometry toolboxes. Then the implemented solution is evaluated using a simulated cuboidal test environment of 11.00 m × 7.50 m × 4.00 m size. The space is sampled with a constant step of 50.00 cm in three directions resulting in a test grid of samples. The sampling coordinates located on the border of the workspace, which physically would be placed on the walls, are excluded from the sampling grid.

The RTLS consists of four LSs, two located in the workspace corners on height 4 m and two located on height 3 m, see Table 1. The origin of the coordinate system is arbitrarily located on the floor at the workspace corner under LS_A_. The calibration point is located approximately in the centre of the workspace, explicitly at 5.60 m × 4.00 m × 1.00 m.

The model is applied to estimate the map of location uncertainty in terms of precision for two pairs of LSs, *AB* and *AD*, located on the longer and shorter walls of the test room respectively. For each sampling point, the location precision expressed as a volume is calculated from the azimuth and elevation AoPs. The SEM of azimuth and elevation AoAs for the tested workspace were heuristically determined from results of tests performed on 36 arbitrary selected location points of workspace for all LSs. The heuristically estimated mean SEM values of both azimuth and elevation AoAs’ standard deviations were 0.45°. Therefore, the measurement uncertainty in terms of precision of each LS is represented by a cone with vertices in LSs’ positions, the axes are defined as the arrival path (Section 5.2) and the circular base with the radius calculated using Equation (13) or (14). The modelled localization precision was determined by using the common volume of two cones, whose axes intersect at the sample location. The localization precision was calculated using the 3D Delaunay triangulation computing method (Section 6.2).

**Figure 7:**
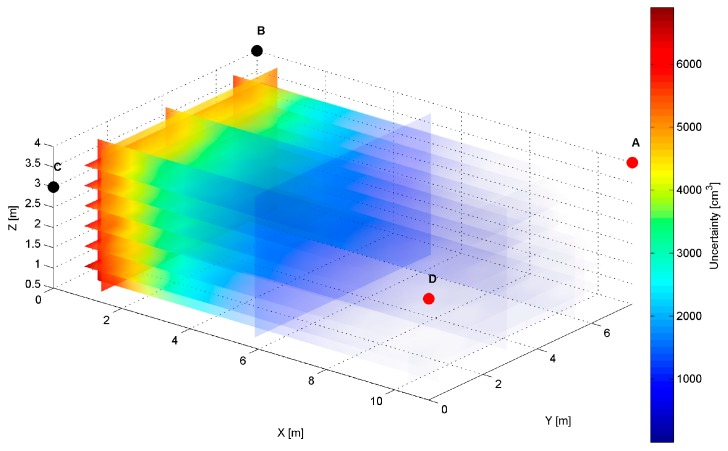
Volumetric slice plot of uncertainty-precision distribution for location sensors *AD*.

The distribution map of the modelled AoA localization precision for an *AD* pair is presented in Figure 7. The LS_A_ and LS_D_ are located at slightly different heights of the shorter wall of the workspace, as shown in Table 1. The slices represent orthogonal planes through the volume of the workspace, and the colours correspond to precision levels.

**Figure 8:**
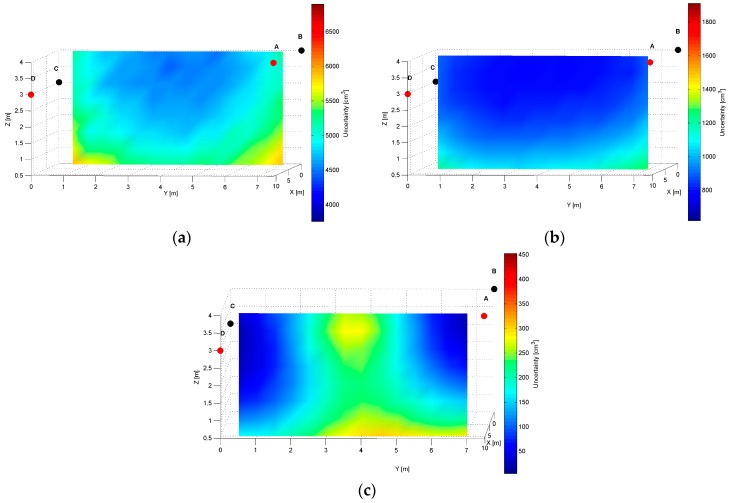
Cross-section (*YZ* plane) plots of uncertainty-precision distribution for location sensors *A* and *D* at (**a**) *x* = 1.00 m; (**b**) *x* = 5.50 m; (**c**) *x* = 10.00 m. All plots are in different colour scales.

**Figure 9:**
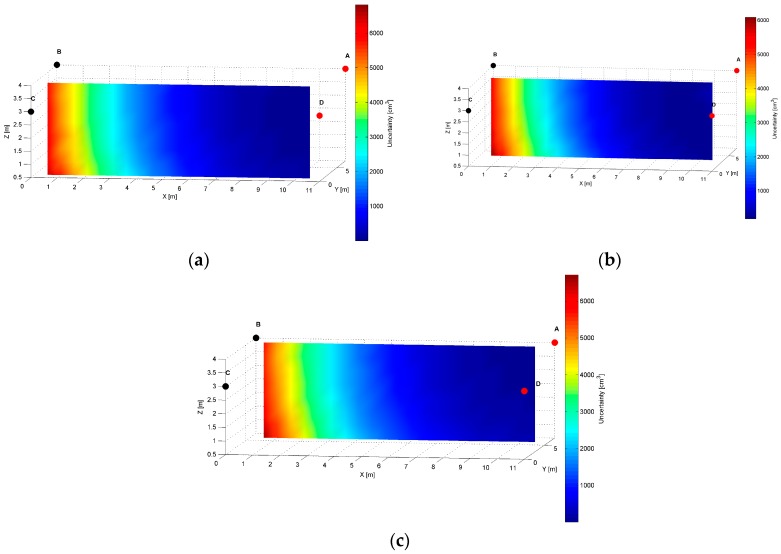
Cross-section (*XZ* plane) plots of uncertainty-precision distribution for location sensors *A* and *D* at (**a**) *y* = 1.00 m; (**b**) *y* = 3.50 m; (**c**) *y* = 6.00 m. All plots are in different colour scales.

**Figure 10:**
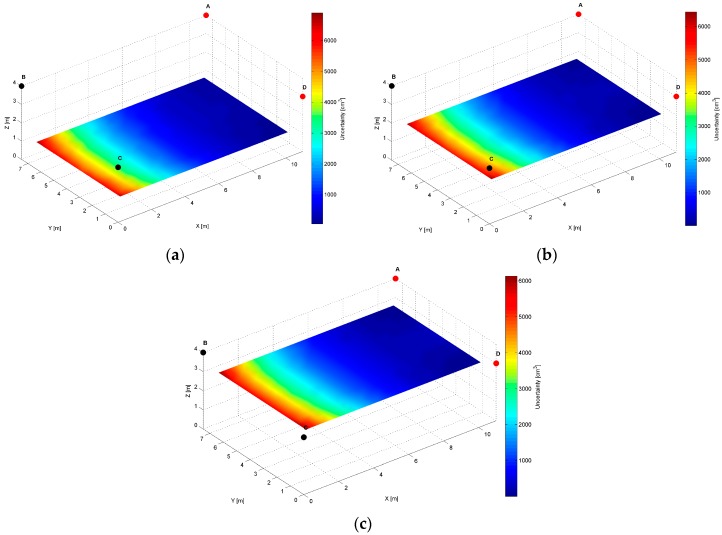
Cross-section (*XY* plane) plots of uncertainty-precision distribution for location sensors *A* and *D* at (**a**) *z* = 1.00 m; (**b**) *z* = 2.00 m; (**c**) *z* = 3.00 m. All plots are in different colour scales.

The distinct cross-sections of the AoA measurement precision distribution maps for the *AD* pair are presented in Figure 8, Figure 9 and Figure 10. Red dots represent the positions of active LSs and black dots show locations of inactive LSs. From the maps, it can be seen that the precision significantly depends on the distance from LSs. For the *AD* pair, located on the shorter room wall, the uncertainty increases along with the distance from LSs. At the bottom corners of the workspace, on the opposite side to the active sensors, the uncertainty is the highest, see Figure 8 and Figure 9. The best precision is identified near the active LSs, which is depicted in Table 2. The precision range is from 150 cm^3^ to 6900 cm^3^. The effect of the LSs’ deployment on different heights is noticeable in Figure 8c, which illustrates the precision distribution at a distance 1 m from the wall where the active sensors are located. Figure 8c shows that the uncertainty level near the floor (*z* = 0.5 m) is lower around the LS_D_ of approximately 170 cm^3^, compared to the area around the LS_A_ where the uncertainty reaches 270 cm^3^.

**Figure 11:**
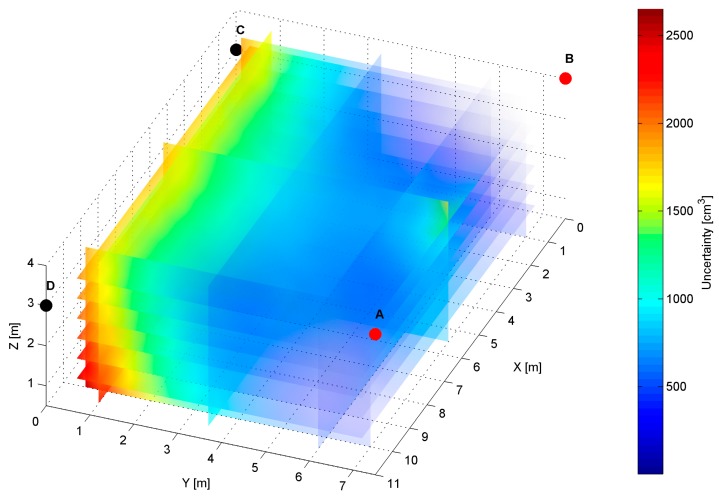
Volumetric slice plot of uncertainty-precision distribution for location sensors *A* and *B*.

For the *AB* pair, located at the same height of the longer room wall, see Table 2, evaluated AoA precision distribution map, is presented in Figure 11.

**Figure 12:**
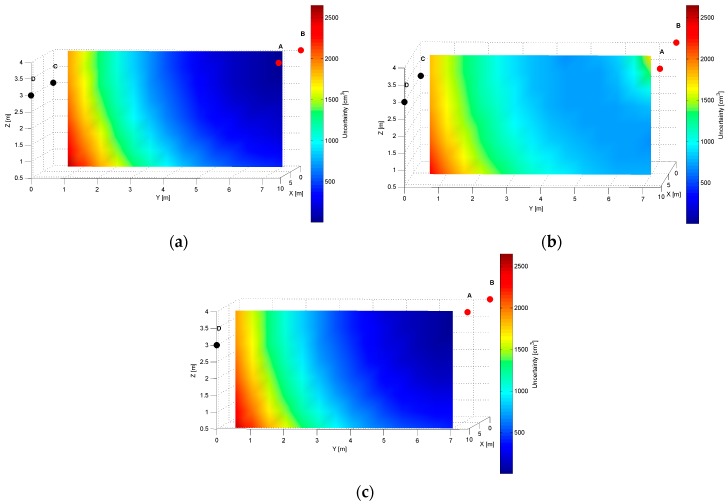
Cross-section (*YZ* plane) plots of uncertainty-precision distribution for location sensors *A* and *B* at (**a**) *x* = 1.00 m; (**b**) *x* = 5.50 m; (**c**) *x* = 10.00 m.

**Figure 13:**
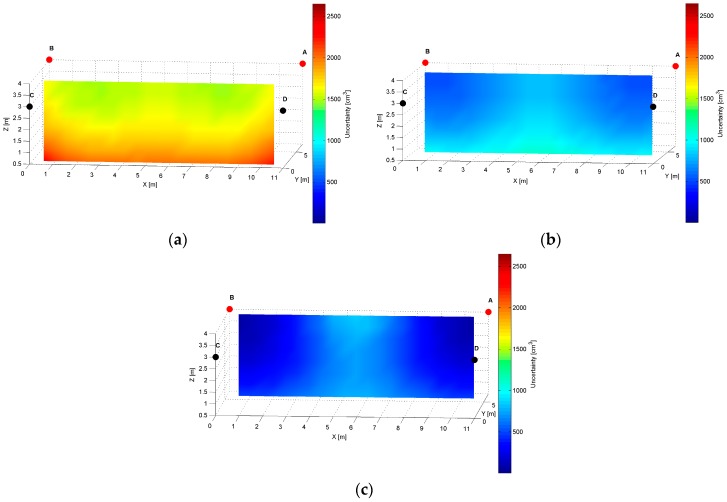
Cross-section (*XZ* plane) plots of uncertainty-precision distribution for location sensors *A* and *B* at (**a**) *y* = 1.00 m; (**b**) *y* = 3.50 m; (**c**) *y* = 6.00 m.

**Figure 14:**
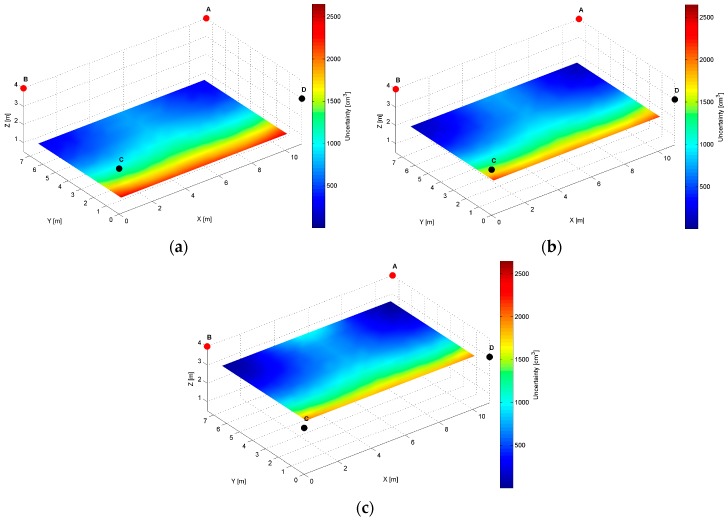
Cross-section (*XY* plane) plots of uncertainty-precision distribution for location sensors *A* and *B* at (**a**) *z* = 1.00 m; (**b**) *z* = 2.00 m; (**c**) *z* = 3.00 m.

The specific cross-sections of the localization precision distribution map for the *AB* pair are presented in Figure 12, Figure 13 and Figure 14. As for the LS’s pair *AD,* also for the *AB* pair, the localization precision increases along with the distance from LSs, see Figure 12 and Figure 14. The worst precision of 2650 cm^3^ is depicted at the bottom corners most distant from the active LS’s *AB* pair. The best precision equals 20 cm^3^, which is noticed at (10.5, 7, 3.5) and (0.5, 7, 3.5) coordinates near the active LSs, see Figure 14a and Table 2. At the top along the wall between LS_A_ and LS_B_, the level of precision is relatively higher, because the cones’ axes referring to the arrival path could be even parallel, which is shown in Figure 12b.

## 8. The Test Systems

**Figure 15:**
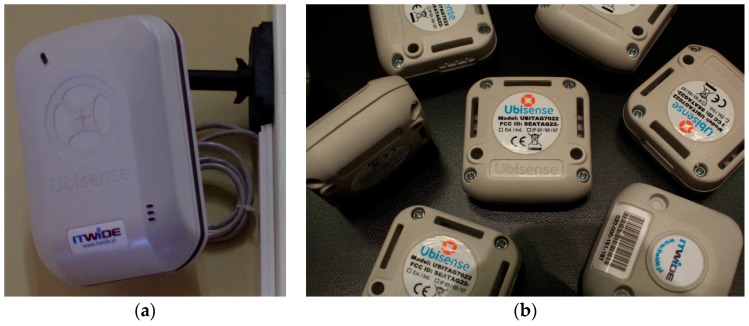
Photos of (**a**) RTLS sensor; (**b**) RTLS tags.

The real system was implemented on the Ubisense Real Time Location System Series 7000. The used UWB-based RTLS consists of four LSs in a master-slave configuration, see Figure 15a. One of the LSs is assigned as a master, which has two-way communication with a tag in the 2.4 GHz telemetry channel. All LSs, both master and slaves, are able to receive localization pulses on UWB channel 6 GHz–8 GHz from the tag as shown in Figure 15b. Also all the LSs communicate by timing and Ethernet connections. Timing connections are used to synchronize the slaves with the master for TDoA measurement. Via Ethernet, the collected raw data is transmitted between the LSs and the switch. It is also used to provide power supply for the LSs according to PoE standards. The switch is connected with the PC consisting of the location estimation platform Ubisense Location Platform 2.1.

The used tags are Ubisense Compact Tags with a maximum tag update rate of 33.75 Hz. The tags attached to the targets communicate with the master on the telemetry channel 2.4 GHz, and send UWB chirps to all LSs. An exemplary structure of UWB-based RTLS is presented in [4]. Each tag sends UWB pulses to all LSs with a defined update rate, which depends on the number of tags in the system. Localization pulses received by each LS are analysed in terms of:

- time difference of arrival to the LSs i.e., TDoA;
- angles at which the signals are received by the LSs in terms of azimuth and elevation AoAs.

The power of signal, RSS, is used by the system for data filtration. The location estimation platform provides various static and dynamic filters, which improve estimation quality. However, in the following physical experiment, the proposed AoA-based localization algorithm operates on AoA raw measurements without filtration. The Ubisense algorithm is a part of the location estimation platform and it was used in validation. The Ubisense hybrid algorithm estimates the tag location based on AoA and TDoA measurements with static filtering.

**Figure 16:**
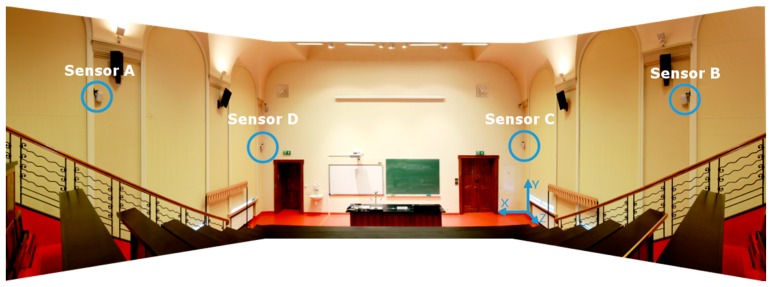
Photo of the indoor environment with mounted LSs and the coordinate system origin.

The physical experiment was performed in a lecture hall with a size of 11.0 m × 10.0 m × 7.0 m located in the over 100 year old building of Faculty of Electrical and Control Engineering in Gdansk University of Technology, illustrated in Figure 16. However, due to the hall’s shape, the RTLS’s workspace does not cover the whole hall’s space. To cover the whole workspace, two additional LSs would be needed. Therefore, the effective size of the workspace is only 11.0 m × 7.0 m × 4.0 m. In the workspace, two environments can be specified, the stage with the lecture hall rostrum, and the tiered seating with 6 rows of desks along with 12 seats.

In the workspace, nine reference points on three desks were established using an electronic tachymeter. Two reference points were defined at the edges and one in the middle of each desk row. The calibration point located approximately in the centre of the workspace at coordinate (5.59, 3.96, 1.06), in the middle of the second desk row, was also one of the reference points.

The LSs *CD*, presented in Figure 16, were installed in the corners of the lecture hall on height 3 m. The two other LSs, *A* and *B*, were placed in the middle of sidewall, on height 3.9 m, see Table 1. All LSs were directed to the calibration point. The LSs positions were established using an electronic tachymeter South NTS-372 RC.

Four different tags, as shown in Figure 15b, were successively mounted on a tripod at four adjustable heights of 0 cm, 30 cm, 60 cm, and 90 cm. The measurements were performed on surfaces of the first, third and fifth desk rows, where each desk row has a different height relative to the floor. The tripod was placed successively at all reference points, at nine *XY* coordinates at four heights. In total, there were 36 spatial location samples with 200 samples at each location of the azimuth and elevation AoAs.

## 9. Experimental Verification

The suitability of the proposed systematic approach to the uncertainty, in terms of accuracy and precision of the AoA-based 3D localization, was verified by physical experiments for *AD* and *AB* LS pairs. The presented localization precision model was verified by evaluating how the model matches the real measurements for these LS pairs. Likewise, the suggested localization offset error model and effects of the correction vector were verified heuristically. Finally, the performance of the AoA-based 3D localization method, including its enhancement, was compared with the reference RTLS’s commercial algorithm results, where the reference localization technique combines the AoA and TDoA localization methods using all LSs.

### 9.1. Precision Model Verification

**Figure 17:**
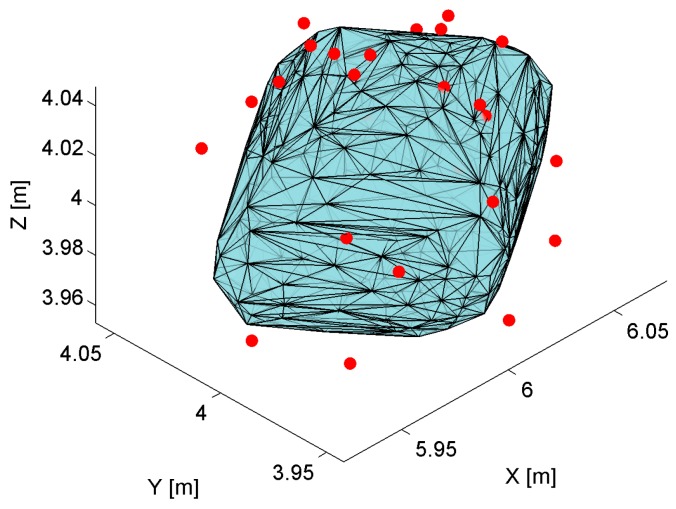
The example of theoretical solid with a number of experimental location estimates outside the solid (red dots).

To verify the localization precision model, the simulation results of the proposed geometrical model of AoA localization in a 3D indoor space were compared with the results of physical experiments. The matching ratio, as a percentage of the experimental location estimates occurring inside the theoretical solid from the model, is used as a quantitative measure of how the precision model fits the reality. The matching ratio was determined at each of the 36 spatial location samples in the workspace, and an example is shown in Figure 17.

**Figure 18:**
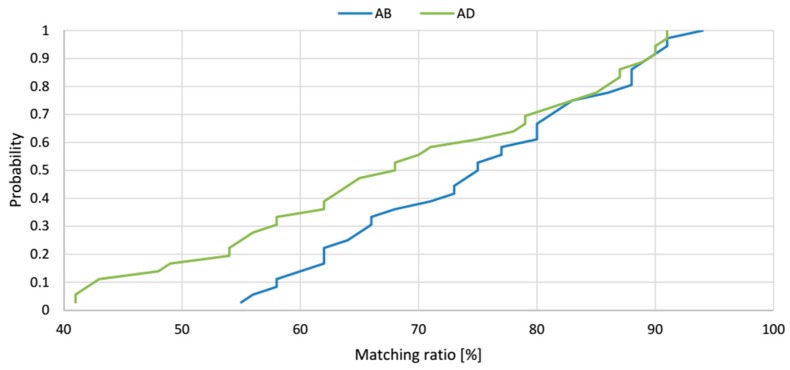
The cumulative distribution functions of the matching ratios for *AB* and *AD* pairs.

Cumulative distribution functions of matching ratios at 36 spatial location samples for LSs *AB* and *AD* are presented in Figure 18. The matching ratio for the pair *AB*, varies from 55% to 94% and the average value of matching ratio for these 36 spatial location samples is 74.6%. The cumulative distribution function for the pair *AD* located on the shorter side of the workspace shows that the matching ratio varies from 41% to 91%, and the average value of matching ratio for the 36 spatial location samples is 68.4%. The *AB* pair of LSs placed on the longer side of the workspace shows better performance and even better fitting of the model to the real measurements. Moreover, for the 50% test points, the matching ratio was bigger than 68% for the *AD* pair and bigger than 75% for the *AB* pair of LSs. The cumulative distribution functions shown in Figure 18 verify that the results of the theoretical model and experiments are consistent.

### 9.2. Validation of Localization Offset Error Approach

The validation of the proposed localization offset error approach is based on an analysis of the tags’ locations estimated using the AoA-based localization algorithm at the 36 spatial location samples in the test environment for the *AB* and *AD* LSs pairs. At each spatial location sample with known coordinates, each tag’s location was estimated from 200 samples. Then, the average offset error of *x*, *y* and *z* components and the resultant average offset error of the location system in the tested environment were computed using Equations (1) and (2) and are shown in Table 3 and Table 4 for *AB* and *AD* pairs, respectively. The estimated average offset error of the *x*, *y* and *z* components defined a correction vector expressed by Equation (12) and calculated using Equation (11). The correction vector was applied to each of the 36 spatial location samples, by subtracting the vector’s coordinates from the estimated average *x*, *y* and *z* localization coordinates. As result of applying the correction vector, the mean offset error components for these 36 spatial location samples were reset to zero, see Table 3 and Table 4.

To judge the correction effect, the new estimated localizations were compared with the reference RTLS’s localization results as shown in Table 3 and Table 4 for *AB* and *AD* LSs pairs respectively. The reference results were obtained using the commercial hybrid algorithm provided by Ubisense, which estimated the tag’s location based on TDoA measurements from four LSs [4].

#### 9.2.1. AB Pair Case Study

**Figure 19:**
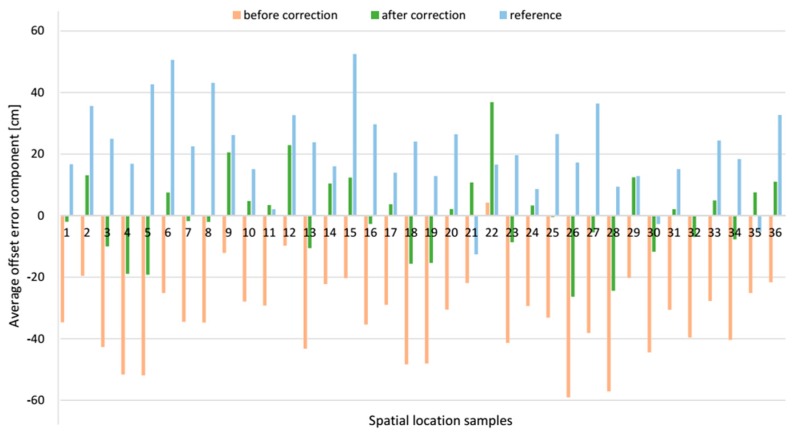
Comparative bar plots of the average offset errors of the *z* components for *AB* pair.

**Figure 20:**
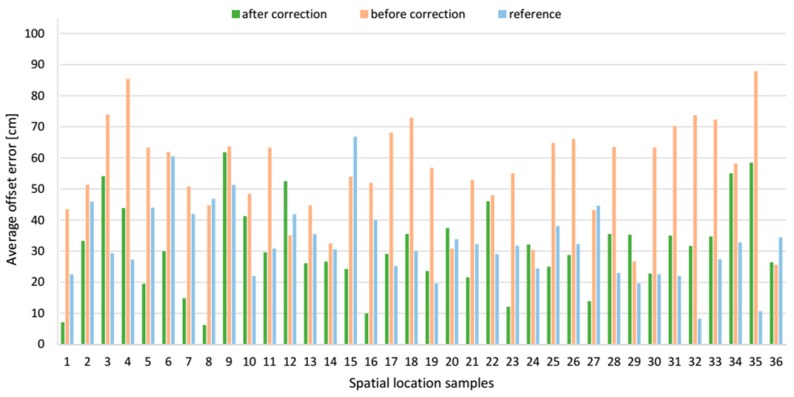
Comparative bar plots of the average resultant offset error for *AB* pair.

The exemplary *z* offset error component, before and after applying the correction vector in each spatial location sample, is compared with the corresponding component from the reference system, see Figure 19. The resultant location offset errors, which are the modules of the relevant offset error vector are shown in Figure 20. After applying the correction vector, the mean resultant offset error for the AoA-based localization algorithm is reduced 43.8% from 55.5 cm with SD of 15.6 cm to 31.2 cm with SD of 13.9 cm see Table 3. For a comparison, the mean resultant offset error determined by the reference algorithm was 32.8 cm with SD of 12.2 cm. Moreover, the correction vector significantly reduces the minimum and maximum values of the resultant offset error by 19.3 cm and 26.1 cm respectively, which means 75.4% and 29.7% respectively. For a comparison, the range of resultant offset error determined by the reference algorithm was from 8.3 cm to 66.8 cm.

The presented data for the *AB* pair shows that for both localization algorithms, the *z* component of the offset error was biggest compared to *x* and *y* components, see Table 3. For the AoA-based localization algorithm without correction vector, *z* component’s mean value was −32.7 cm and standard deviation, SD, 13.4 cm, when for the reference algorithm *z* component’s mean value and SD were 20.7 cm and 14.5 cm respectively. For this LSs pair, the *x* component has the least impact on the resultant offset error. For both algorithms, the mean offset error *x* component was almost a half of *z* component.

**Figure 21:**
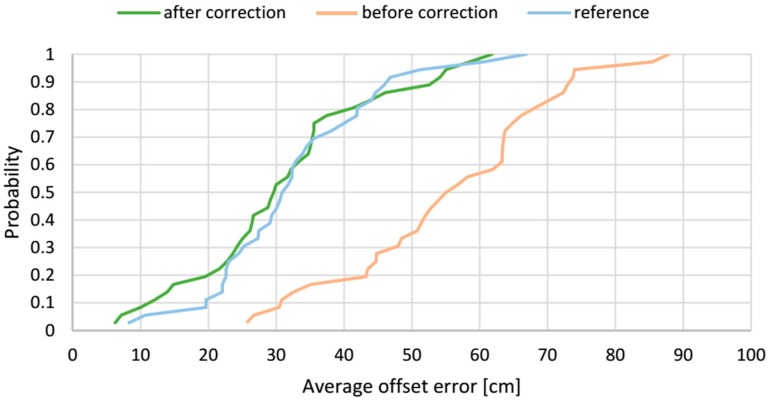
Experimental cumulative distribution functions of the average resultant offset error for LSs *AB* pair for three algorithms.

The cumulative distribution functions of average offset errors from tests of AoA-based localization method before and after correction along with the reference method are presented in Figure 21. The presented plots show how the correction vector influences the average offset error. In a case the AoA-based localization algorithm without correction, the range of the error is from 25 cm to 87 cm and the median of the average offset error is 55 cm. The correction vector significantly shifts the distribution function to the left to the range from 6.3 cm to 61.8 cm with a median of 30 cm. For 80% of test locations, the average offset error, after applying the correction vector, is less than 40 cm.

#### 9.2.2. AD Pair Case Study

**Figure 22:**
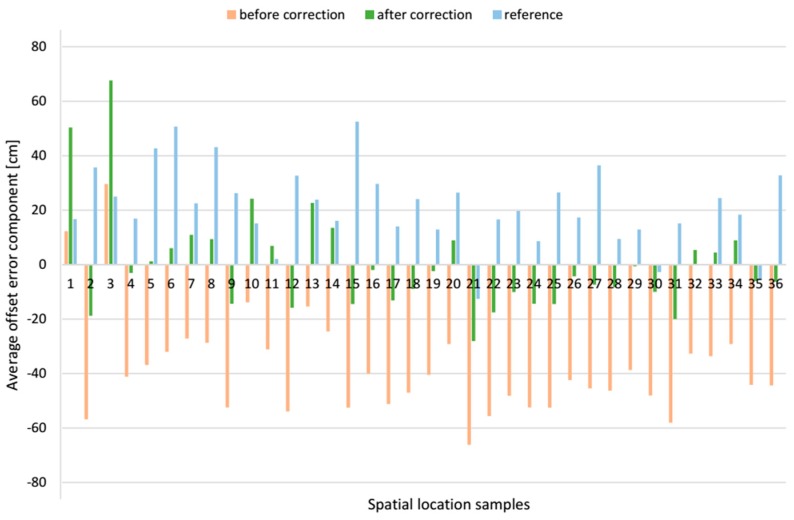
Comparative bar plots of the average offset errors of the *z* components for *AD* pair.

**Figure 23:**
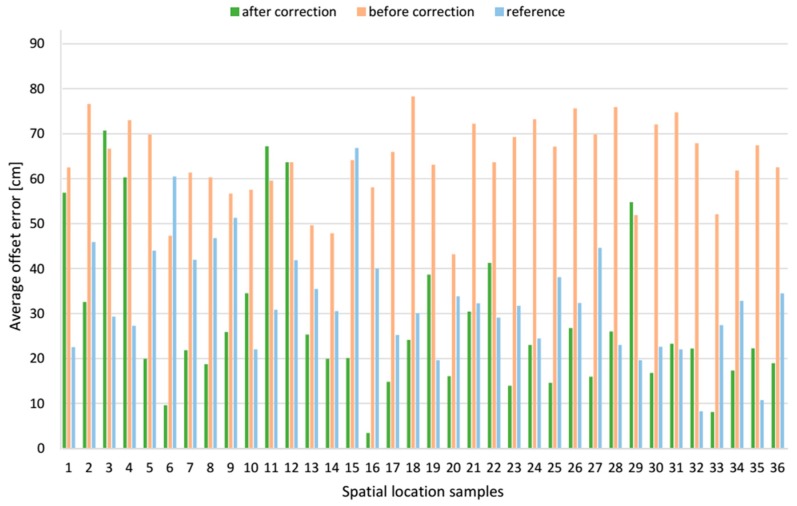
Comparative bar plots of the average resultant offset error for *AD* pair.

The *z* offset error components before and after applying the correction vector at each spatial location sample are compared in Figure 22, and the resultant location offset errors are shown in Figure 23. It shows that the correction vector reduced the location offset errors at most of the examined positions, and the average offset error of these 36 locations is reduced 55.6% from 64.0 cm to 28.4 cm, see Table 3. However, the SD of mean offset error increased from 8.9 cm to 17.1 cm. For a comparison, the mean resultant offset error determined by the reference algorithm was 32.8 cm with the SD of 12.2 cm. Moreover, the correction vector significantly reduces the minimum and maximum values of the resultant offset error by 39.7 cm and 7.6 cm respectively to the range from 3.5 cm to 70.7 cm, what means improvement of 91.9% and 9.7% respectively. For a comparison, the range of resultant offset error determined by the reference algorithm was from 8.3 cm to 66.8 cm.

The presented data for *AD* LSs pair show that alike for the *AB* LSs pair, the *z* component of the offset error was the biggest, compared to *x* and *y* components. For AoA-based localization algorithm without the correction vector, *z* component’s mean value was −38.1 cm with a SD of 18.6 cm. When for the reference method, *z* component’s mean value was 20.7 cm with a SD of 14.5 cm. The *y* component with the mean value −24.7 cm with a SD of 23.9 cm is the smallest of the three components. However, the SD of this component is relatively high compared to the *x* and *z* components.

**Figure 24:**
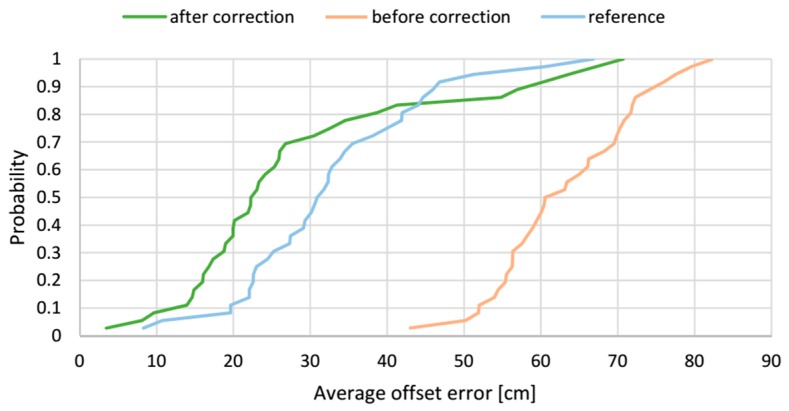
Experimental cumulative distribution functions of the average resultant offset error for LSs *AD* pair.

The cumulative distribution functions of the average offset errors of the AoA-based localization method before and after correction along with the reference method results for the 36 spatial location samples are presented in Figure 24. The presented distribution functions clearly illustrate how the correction vector influences the average offset error. For the results from the AoA-based localization algorithm without the correction vector, the median of the average offset error is 60 cm, whereas the range of the error is from 42 cm to 82 cm. The correction vector significantly shifts the distribution function to the left and the median of average offset error is reduced 63.3% to 22 cm. For 80% of the test locations, the average offset error is less than 40 cm.

## 10. Results Discussion

The simulation results for two LS pairs *AD* and *AB* shown in Figure 7, Figure 8, Figure 9, Figure 10, Figure 11, Figure 12, Figure 13 and Figure 14 indicate that the localization uncertainty, in terms of precision, depends on the LS’s configuration in the workspace. The precision distribution maps demonstrate how the uncertainty increases along with the distance from LSs; the uncertainty is worst at the bottom corners of the side opposite to the side with the active LSs, which is summarized in Table 2.

The Table 2 points out that the best precision is identified near the active LSs. The simulation results indicate also how different deployment heights of active LSs influence the uncertainty maps. For instance, Figure 8c shows that the location uncertainty near the floor is significantly lower under the LS_D_, which is located lower than the LS_A_. The simulation results of the LSs *AB* pair placed at the same height show a relatively high uncertainty at the middle top along the wall between LS_A_ and LS_B_, see Figure 12b. The reason for this phenomenon is that in this area, the volume of the common solid of the two crossing cones is relatively big since their axes referring to the paths of arrival could be almost coaxial. However, the cones are not coaxial due to the established 50 cm localisation dead zone near the wall. To overcome a problem of bad uncertainty-precision near the wall, it can be suggested to arrange the active LSs at different heights.

The simulated uncertainty maps for the *AB* and *AD* LS pairs depict the advantage of the *AB* pair, which provide the localization precision in a range from 20 cm^3^ to 2650 cm^3^ compared to *AD* pair’s a range from 150 cm^3^ to 6900 cm^3^, see Table 1. The verification of this observation by the experimental results confirms that the best localization precision is achieved for the LSs *AB* pair and consequently for *AD* pair, the average value of matching ratio for the 36 spatial location samples is 74.6% compared to 68.4% for the LSs *AD* pair.

The customization verification proves a positive influence of the correction vector on localization accuracy. In the given test environment with 36 test points, for LSs *AB* pair, the correction vector reduces 55% the mean offset error from 55 cm to 31 cm and from 64 cm to 28 cm for *AD* pair, see Table 3 and Table 4. Also for the *AB* pair the ranges of the offset error at these 36 test points have been diminished of 19.3 cm and 26.1 cm for lower and upper range limits respectively, from 25.6 cm and 87.9 cm to 6.3 cm and 61.8 cm respectively. The customization results challenges the mean offset error value of the reference algorithm of 32 cm, and even its range limits of 8.3 cm and 66.8 cm.

To comprehensively assess the correction vector’s effect, the estimated localizations’ accuracy without and with the applied correction vector are compared with the reference RTLS’s results for LSs AB and AD pairs, shown in the Figure 21 and Figure 24, respectively. The figures clearly indicate improvement of the accuracy after applying the correction vector, which vitally moves the cumulative distribution curves towards the lower value of the offset error. Considering both LS configurations, after applying the correction vector, a half of the test locations, the offset error is less than 30 cm and 22 cm for *AB* and *AD* pairs respectively, compared to 31 cm for the reference algorithm. One can see that the correction vector improves the accuracy of the AoA method in a way that it challenges the reference method. Similarities in shape of all cumulative distribution functions, including the reference one, can indirectly validate the presented approach.

## 11. Conclusions and Future Work

Due to its applicability and complexity, discovering a trade-off among different features of indoor localization systems working in a 3D environment is an important research subject. The proposed approach investigates the performance in terms of the localization uncertainty of AoA-based UWB-based RTLS’s. The improvement of localization accuracy and precision of the RTLS’s, without compromising its simplicity and price has been achieved by means of the system customization. The proposed analytical geometrical uncertainty model of the AoA localization method in a 3D indoor space is the concept’s foundation of the analytical customization method. The customization is based on the performance assessment in a given working environment.

A 50% improvement of system localization accuracy is gained by applying a correction vector, which is heuristically defined from an analysis of the system’s 3D accuracy distribution map of the given working space. The enhanced performance of the AoA-based UWB-based RTLS challenges the performance of the reference hybrid TDoA methods supported by AoA technology, whereas the proposed method excels the reference one in terms of simplicity and price. The experimental results prove that the correction vector is the suitable customization, which reduces the localization offset error caused by the variety of the system’s architecture and calibration process, and by the tags’ and working environments’ heterogeneity.

Another introduced customization approach considers the system’s performance in terms of precision in respect to the system’s configuration in the given working space. The system’s performance analysis for different LSs configuration was done for two different LSs pairs and for different LSs’ height placement in the space of the lecture hall. The results show a significant difference in precision, up to 7.5 times for its lower limit and 2.6 times for its upper limit, for the two considered configurations. Furthermore, the analysis indicates also a disadvantage of placing the active LSs at the same height. Moreover, the simulated precision distribution maps define the areas of the best and worst localization precision, in such a manner that the best performance is noticeably near to the active LSs and the worst are at the corners hindmost from these LSs.

The angular-based 3D localization algorithm estimating the tag’s location using azimuth and elevation angle measurements of a pair of LSs is proposed. The extrapolation algorithm allows finding the localisation estimate even in contaminated environments by using the principle of a distance between two skewed lines. Simulation and physical experiment results confirm that the proposed simple extrapolation angular-based 3D localization algorithm ensures a good localization performance and challenges the advanced UWB-based RTLS algorithms.

The proposed analytical geometrical model of the AoA localization method in a 3D indoor space was evaluated in the simulation environment of Matlab where the model was implemented. The proposed solution was verified by comparison of simulation and physical experiment results. The quantitative verification, in a form of matching ratios confirms that the analytical model matches the real measurement with a high probability level.

To further enhance the RTLS performance, the research may consider a region-based correction vector method, which adjusts the correction vector to the region of workspace. The distance from the active LSs can be used as an adaptive factor of the correction vector. The artificial intelligent approach, such as fuzzy logic or machine learning, may be implemented for estimation of regions’ boundaries and relevant suitable correction vectors. Additionally, the uncertainty analysis of the UWB LS’s array geometry used in the AoA-based RTLSs may provide guidance on how to enhance the estimation of the correction vectors.

Moreover, a similar localization uncertainty analysis can be applied to ToA and TDoA-based localization algorithms used in RTLSs. An analogical uncertainty model, including offset error sources of RTLS’s architecture, indoor environment and synchronization procedure, can be defined to enhance the system’s performance.

## Figures and Tables

**Table 1:** Sensors’ arrangement in simulation and physical experiment.

LS	Simulation (*x, y, z*) (m)	Physical Experiment (*x, y, z*) (m)
LS_A_	(11.00, 7.50, 4.00)	(11.03, 7.42, 3.88)
LS_B_	(0.00, 7.50, 4.00)	(0.17, 7.38, 3.89)
LS_C_	(0.00, 0.00, 3.00)	(0.17, 0.17, 3.01)
LS_D_	(11.00, 0.00, 3.00)	(11.00, 0.17, 2.92)

**Table 2:** Simulated extreme uncertainty values for *AB* and *AD* pairs.

Pair of LSs	Best Precision		Worst Precision	
	Value (cm^3^)	Location	Value (cm^3^)	Location
*AB*	20	corner of the LS_A_	2650	ground corner below LS_C_
		corner of the LS_B_		ground corner below LS_D_
*AD*	150	corner of the LS_A_	6900	ground corner below LS_B_
		corner of the LS_D_		

**Table 3:** Characteristics of the experimental results of the offset error for the 36 spatial location samples for the AoA-based localization algorithm before and after correction and the reference algorithm for LS’s *AB* pair.

Component	Offset Error for the AoA-Based Localization Algorithm						Offset Error for the Reference Method		
	Before Correction			After Correction					
	Mean (cm)	SD * (cm)	Range (cm)	Mean (cm)	SD * (cm)	Range (cm)	Mean (cm)	SD * (cm)	Range (cm)
x	−15.9	23.4	−72.0–38.8	0	23.4	−56.2–54.7	−11.4	6.1	−26.4–1.34
y	−29.0	20.9	−67.6–21.7	0	20.9	−38.6–50.7	−12.0	15.9	−39.2–43.4
z	−32.7	13.4	−59.1–4.2	0	13.4	−26.4–36.9	20.7	14.5	−12.6–52.5
Resultant	55.5	15.6	25.6–87.9	31.2	13.9	6.3–61.8	32.8	12.2	8.3–66.8

* SD of the mean offset error of 36 spatial location samples.

**Table 4:** Characteristics of the experimental results of the offset error for the 36 spatial location samples for the AoA-based localization algorithm before and after correction, and the reference algorithm for LS’s *AD* pair.

Component	Offset Error for the AoA-Based Localization Algorithm						Offset Error for the Reference Method		
	Before Correction			After Correction					
	Mean (cm)	SD * (cm)	Range (cm)	Mean (cm)	SD * (cm)	Range (cm)	Mean (cm)	SD * (cm)	Range (cm)
x	−31.8	13.4	−58.3–5.0	0	13.4	−26.4–36.9	−11.4	6.1	−26.4–1.34
y	−24.7	23.9	−51.1–42.1	0	23.9	−26.4–66.8	−12.0	15.9	−39.2–43.4
z	−38.1	18.6	−66.1–29.6	0	18.6	−28.1–67.7	20.7	14.5	−12.6–52.5
Resultant	64.0	8.9	43.2–78.3	28.4	17.1	3.5–70.7	32.8	12.2	8.3–66.8

* SD of the mean offset error of 36 spatial location samples.

## Abbreviations

The following abbreviations are used in this manuscript:

AoA	Angle of Arrival
AoP	Angle of Precision
DoP	Dilution of Precision
GDoP	Geometric Dilution of Precision
ILS	Indoor Localization System
IMU	Inertial Measurement Unit
LoS	Line-of-sight
LS	Location Sensor
NLoS	Non-line-of-sight
PoE	Power over Ethernet
RFID	Radio Frequency Identification
RSS	Received Signal Strength
RTLS	Real-time Locating Systems
SD	Standard Deviation
TDoA	Time Difference of Arrival
ToA	Time of Arrival
UWB	Ultra Wideband
